# Evidence of linkage to chromosome 1 for early age of onset of rheumatoid arthritis and HLA marker DRB1 genotype in NARAC data

**DOI:** 10.1186/1753-6561-1-s1-s78

**Published:** 2007-12-18

**Authors:** Wei Xu, Hui Lan, Pingzhao Hu, Shelley B Bull, Celia MT Greenwood

**Affiliations:** 1 Department of Biostatistics, Princess Margaret Hospital, Toronto, Ontario, 610 University Avenue, Room 15-507, Toronto, Ontario, Canada M5G 2M9; 2 Department of Public Health Sciences, University of Toronto, 6^th ^Floor, Health Science Building, 155 College Street, Toronto, Ontario, Canada M5T 3M7; 3 Genetics and Genomic Biology, Hospital for Sick Children, TMDT Building, 101 College Street, Room 15-706, Toronto, Ontario, Canada M5G 1C7; 4 Department of Computer Science, University of Toronto, Stanford Fleming Building, 10 King's College Road, Room 3302, Toronto, Ontario, Canada M5S 3G9; 5 Samuel Lunenfeld Research Institute, Mount Sinai Hospital, Suite 5-226, 60 Murray Street, Toronto, Ontario, Canada M5G 1X5

## Abstract

Focusing on chromosome 1, a recursive partitioning linkage algorithm (RP) was applied to perform linkage analysis on the rheumatoid arthritis NARAC data, incorporating covariates such as HLA-DRB1 genotype, age at onset, severity, anti-cyclic citrullinated peptide (anti-CCP), and life time smoking. All 617 affected sib pairs from the ascertained families were used, and an RP linkage model was used to identify linkage possibly influenced by covariates. This algorithm includes a likelihood ratio (LR)-based splitting rule, a pruning algorithm to identify optimal tree size, and a bootstrap method for final tree selection.

The strength of the linkage signals was evaluated by empirical *p*-values, obtained by simulating marker data under null hypothesis of no linkage. Two suggestive linkage regions on chromosome 1 were detected by the RP linkage model, with identified associated covariates HLA-DRB1 genotype and age at onset. These results suggest possible gene × gene and gene × environment interactions at chromosome 1 loci and provide directions for further gene mapping.

## Background

Rheumatoid arthritis (RA) is a chronic, inflammatory autoimmune disease in which the patient's immune system attacks primarily the joints. The mean age of onset is between 45 and 50 years of age, although it can occur at any age; its prevalence may be as high as 1% in adults [1]. The etiology of RA remains unknown. Many studies have shown that RA has strong association with the HLA marker DRB1 and there may be other genetic factors [2,3]. This disease preferentially affects women (it is three times more common in women than men). Aside from genetic factors and sex, other environmental factors such as smoking confer about a two-fold increased risk.

Our primary goal is to improve the understanding of the etiology of RA through more detailed linkage analysis. It is interesting to know to what extent covariates such as smoking, sex, or age at onset can influence the identification of genetic loci that predispose for RA. Previous studies have found linkage evidence on chromosome 1 [4-6], with three regions (1q36.21, 1q32.1, 1q44) implicated in Caucasian RA patients. However, these studies have not identified environmental factors or gene × gene interactions influencing linkage on this chromosome.

Some existing model-free linkage analysis methods that allow covariates can incorporate only one or few covariates and rely on an assumption of linear covariate effects [7-9]. However, for the NARAC data, 10 covariates were collected for each affected family member including sex, age at onset, HLA-DRB1 genotype, severity, anti-cyclic citrullinated peptide (anti-CCP), smoking, race, rheumatoid factor (RF), subcutaneous nodules, and joint alignment and motion score (JAM). Several covariates may jointly affect the identity-by-descent (IBD) allele-sharing pattern of linked genetic markers in a nonlinear way. In this study, we implemented a method for simultaneously testing for linkage while incorporating possible covariates that are associated with the linkage measurement at that locus [10,11]. We have previously shown that this strategy can improve power to detect linkage in the presence of gene × environmental interaction [11]. The method thus provides additional information to improve understanding of disease etiology. Our objective is to apply this method to chromosome 1 to identify genetic markers linked to disease susceptibility genes combination with environmental factors or with the HLA gene.

## Methods

### Statistical model

We applied the method of Xu et al. [10] for the analysis of affected-relative-pair (ARP) data to detect linkage in the presence of gene × environment interactions. This method, recursive partitioning linkage (RP), is a tree-based model for linkage analysis allowing covariates such as truly environmental factors (e.g., air pollution), demographic factors (e.g., sex, ethnicity, age), or genotypes at other loci.

The RP algorithm uses log-likelihood ratio (LLR) statistics for constructing a splitting rule based on a likelihood-ratio (LR)-based linkage model [9]. Pair-level covariates are used for sample splitting in the RP model. A likelihood ratio test statistic for linkage can be written as:

$$LLR=\sum_{p=1}^{N}\log\left(\frac{\sum_{i=0,1,2}\lambda_{i}g_{ip}}{\sum_{i=0,1,2}\lambda_{i}f_{i(p)}}\right),$$

where the LLR is summed over all the *N *affected sib pairs. The parameter *λ*_*i *_measures the excess risk to an individual who shares, at the marker locus, *i *alleles IBD with an affected sibling compared to the population risk. *λ*_1 _corresponds to IBD = 1, *λ*_2 _corresponds to IBD = 2, and *λ*_0 _= 1·*f*_*i*(*p*) _is the prior probability of sharing *i *alleles IBD for affected sib pair *p*·g_*ip *_represents the estimated probabilities of sharing *i *alleles IBD based on marker data for pair *p*. The parameters *λ*_*i *_are estimated by optimizing the total LR for all ARPs.

For each pair-defined binary covariate *X*_*p *_(*X*_*p *_= 1 or 2), the LLR of sub-nodes can be used to test linkage in the presence of heterogeneity by estimating two sets of parameters (***λ***_{*Xp *= 1}_, ***λ***_{*Xp *= 2}_). The splitting rule is defined by identification of the covariate that gives the largest LLR statistic over the sub-nodes, that is identifying the strongest linkage heterogeneity. This is implemented recursively to grow a full tree.

The next step consists of a pruning algorithm that trims the full tree, which may otherwise overfit the data. In this study, we used a bootstrap algorithm to estimate the deviance function for choosing the optimal tree size (total number of terminal nodes). The optimal tree size is selected as the one with the largest estimated deviance function [11].

After choosing the optimal tree size, we used an independent bootstrap algorithm to choose the final tree structure. Across the trees generated in bootstrap samples that have the same tree size as the optimal tree size, for each locus, the proportion of the trees with particular covariate splits can be calculated. When one covariate clearly defines linkage heterogeneity, most bootstrap data sets will select that covariate. When several covariates are associated with the disease gene, bootstrap data sets may choose a variety of tree structures and covariates. The RP model chooses, as the final tree, the tree structure that appears most frequently among all the bootstrap sampling trees [11].

The linkage test statistic of a final tree is a global linkage test that reflects marginal linkage and genetic heterogeneity. For each marker, this statistic is calculated as:

$$LLR=\sum_{t=1}^{s}\sum_{p\in t}\log\left(\sum_{i=0,1,2}{\hat{\lambda }}_{it}{\hat{f}}_{ip}/\sum_{i=0,1,2}{\hat{\lambda }}_{it}f_{i(p)}\right),$$

where *s *is the final tree size, the summation is over all the terminal nodes, and the parameters ${\hat{\lambda }}_{it}$ are estimated from the chosen final tree structure.

### NARAC data linkage analysis

Use of the North American Rheumatoid Arthritis Consortium (NARAC) data set [12] was approved by the Hospital for Sick Children Research Ethics Board. Our analysis focuses on chromosome 1, which contains 29 genotyped microsatellite markers. The RA disease status of each family member was provided in the data. There was a total of 1097 individuals in 512 families with 617 affected sib pairs used in the RP model. The covariates age at onset, antibody anti-CCP, RF, and JAM score were dichotomized according to their sample median, other covariates such as race, sex, smoking, HLA-DRB1 genotype (*04 allele as risk allele), severity, and subcutaneous nodules were dichotomized according to their natural levels. For each individual-level covariate, two pair-level covariates were generated. All the pair-level covariates are defined as binary variables with concordance for one of the two levels versus other pairings.

After creating all covariates, we examined their frequencies and excluded from analysis covariates in which the rare category occurred with a frequency of 10% or less, since power would be insufficient in such cases; therefore, 19 covariates were considered by the RP model. Genehunter [13] was used to calculate the multipoint nonparametric linkage (NPL) score at each marker, the estimated IBD sharing, and the prior pattern of IBD sharing for each pair of affected siblings.

The global linkage test statistic does not follow an asymptotic *χ*^2 ^distribution [11]. In order to control the type I error, we therefore simulated 2000 data sets under no linkage keeping the same pedigree structure, covariate information, and disease status, using ALLEGRO 1.2C [14]. Then we applied the RP model in these null data sets to obtain empirical null distributions of the global linkage test statistic. After that, we applied the RP model to the original data set to obtain the global linkage test, and compared it to the empirical null distribution to obtain the empirical *p*-values.

## Results

**Figure 1:**
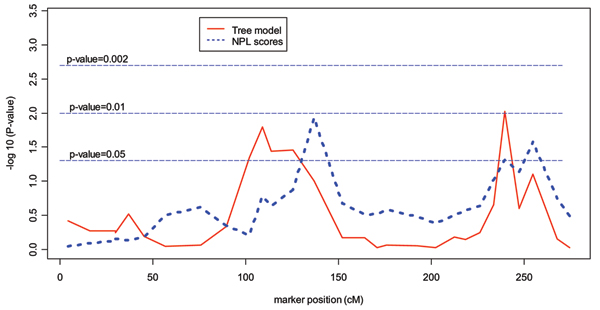
-log10(*p*-values) of the NPL score and of the RP model.

Figure 1 shows -log_10 _of the *p*-values from the NPL scores and the RP model. Neither test provided strong evidence for linkage to chromosome 1, although both methods had minimum *p*-values near 0.01 somewhere on the chromosome. Using the NPL score, a suggestive region was identified near marker 132.66 cM, and using the RP model, we found two other suggestive regions on this chromosome. The first region ranges from 102.02 cM to 125.51 cM, and the second region contains only the F27b marker at 239.66 cM. Table 1 shows the associated covariates in these regions from the RP models.

**Table 1:** Suggestive linkage regions detected by RP model

Marker name (DECODE)	NPL *p*-value	Overall RP asymptotic *p*-value	Overall RP empirical *p*-value	Splitting covariates	Subgroup with largest likelihood ratio	*λ*_1 _and *λ*_2 _for this subgroup
H35a (102.02 cM)	0.62	0.0073	0.045	HLA-DRB1	Both without *04 allele in HLA-DRB1	0.82, 0.35
H19a (109.04 cM)	0.16	0.0014	0.016	HLA-DRB1	Both without *04 allele in HLA-DRB1	0.83, 0.40
F19b (113.69 cM)	0.23	0.0049	0.036	HLA-DRB1	Both without *04 allele in HLA-DRB1	0.84, 0.44
H8c (125.51 cM)	0.13	0.0040	0.035	HLA-DRB1	At least one has *04 allele in HLA-DRB1	1.14, 1.50
F27b (239.66 cM)	0.05	0.0006	0.009	Age at onset	Both age at onset less than median	1.34, 2.23

**Figure 2:**
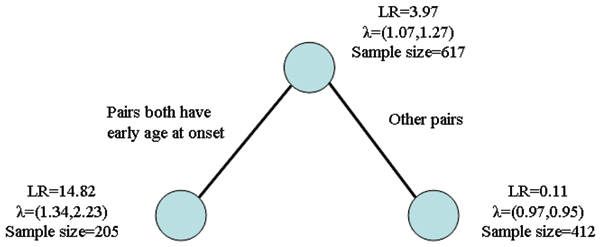
Final tree for marker F27b (*λ *represents the relative risk parameters for affected sib pairs within each node).

The HLA-DRB1 genotype was detected as an associated covariate for all four markers in the first RP-detected region; the high-risk subgroup consisted of relative pairs not carrying HLA-DRB*04. Age at onset was found to be associated with the marker at 239.66 cM (marker F27b). The final tree structure at marker F27b is shown in Figure 2. The LLR of the ASP subgroup concordant for young age at onset (less than the sample median age of 39 years old), is 14.93 with a subset *p*-value is 0.0001; hence this subgroup shows very strong linkage.

## Discussion and conclusion

We applied a recursive-partitioning model for linkage analysis to select covariates that are associated with the allele-sharing patterns in relative pairs. A pruning algorithm based on the bootstrap method and a final tree structure selection algorithm was used to improve the performance of the model. In the NARAC data, we identified two linkage signals involving covariate interactions in regions distinct from the region with the maximum NPL score. However, the NPL peak at 132.66 cM was not identified by the RP model global linkage test. One possible reason is that the necessity of controlling "false" splits in the RP model (through empirical significance levels) may reduce the power to detect marginal linkage signals.

Obtaining empirical significance levels is necessary. We compared the empirical *p*-values of the detected markers to their asymptotic *p*-values based on the *χ*^2 ^distribution, and noted, as expected, that the asymptotic *p*-values were smaller (Table 1). This is due not only to the covariate selection, but also to dependence between pairs from the same pedigree [11].

The HLA_DRB1 gene has been shown to have very strong association with RA [2,3]. Genes in other regions may also be related to RA; however, the linkage signals of those genes may be masked by major gene effects, such as HLA-DRB1 gene. Linkage analysis conditional on genotypes of the major disease genes may improve the power to detect linkage signals in other regions. In this study, the genotypes of HLA-DRB1 were used as potential factors modifying the disease-gene linkage of other RA genes. Our results suggest a possible interaction of the HLA-DRB1 gene and chromosome 1 genes.

The linkage at 239.66 cM was associated with age at onset. This suggests that people with a susceptibility allele in this region have a higher chance to develop RA at an early age. These detected regions and the associated factors provide potential directions for further fine-scale gene mapping.

## Competing interests

The author(s) declare that they have no competing interests.
